# Laparoscopic liver resection of segment 8 via a hepatic parenchymal transection-first approach guided by the middle hepatic vein

**DOI:** 10.1186/s12876-022-02289-8

**Published:** 2022-05-08

**Authors:** Nan You, Ke Wu, Jing Li, Lu Zheng

**Affiliations:** Department of Hepatobiliary Surgery, The Second Affiliated Hospital, Third Military Medical University (Army Medical University), Chongqing, 400037 China

**Keywords:** Laparoscopic liver resection, Liver segment 8, Parenchymal transection-first, Middle hepatic vein

## Abstract

**Background:**

Pure laparoscopic liver resection (LLR) of segment 8 (S8) is still rarely performed due to the lack of an appropriate surgical approach. This article discusses the technical tips and operation methods for LLR of S8 via a hepatic parenchymal transection-first approach.

**Methods:**

Clinical data of 22 patients who underwent LLR of S8 via a hepatic parenchymal transection-first approach guided by the middle hepatic vein (MHV) in the Second Affiliated Hospital, Third Military Medical University (Army Medical University) from May 2017 to February 2020 were retrospectively analyzed.

**Results:**

The mean age was 51.1 ± 11.6 years; mean operation time, 186.6 ± 18.4 min; median blood loss, 170.0 ml (143.8–205.0 ml); and median length of hospital stay, 8.0 days (7.0–9.0 days). There was no case of open conversion. Pathologic findings revealed all cases of hepatocellular carcinoma (HCC). Pathology showed free surgical margins. Post-operative complications included liver section effusion, pleural effusion, pneumonia, intra-abdomen bleeding and bile leak. All the complications responded well to conservative treatment. No other abnormality was noted during outpatient follow-up examination. All patients survived tumor-free.

**Conclusions:**

LLR of S8 is still quite challenging at present, and it is our goal to design a reasonable procedure with accurate efficacy and high safety. We use hepatic parenchymal transection-first approach guided by the MHV for LLR of S8. This technique overcomes the problem of high technical risk, greatly reduces the surgical difficulty and achieves technological breakthroughs, but there are still many problems worth further exploration.

**Supplementary Information:**

The online version contains supplementary material available at 10.1186/s12876-022-02289-8.

## Background

Due to the lack of anatomic landmarks on the liver surface, anatomical variations and cross distribution of intrahepatic Glisson and hepatic venous systems, laparoscopic liver resection (LLR) [1, 2] for lesions in segment 8 (S8) is considered a difficult and challenging surgical procedure by the minimally invasive surgery community. However, with increasing experience and developments in surgical techniques and instruments, limited reports in the last few years have shown the feasibility and safety of this surgery [3, 4]. There are many technical tips for LLR of S8, and the core technical tip is how to choose an appropriate laparoscopic approach, which is a main determinant of surgical success. To date, the approaches for LLR of S8 roughly include ultrasound-guided S8 segmental portal branch puncture, a hepatic hilum Glisson pedicle approach, and a left and right hemi-hepatic splitting approach [5–7]. However, all these approaches have certain drawbacks. Through continuous learning and exploration, we have carried out LLR via a hepatic parenchymal transection-first caudal approach guided by the middle hepatic vein (MHV) and applied it to resection of S8. LLR of S8 via a hepatic parenchymal transection-first approach guided by the MHV is a feasible and effective technique. The specific strategy described here may help laparoscopic surgeons safely perform this challenging procedure.

## Patient and methods

### Patients

The data of patients who underwent laparoscopic liver resectionin the Second Affiliated Hospital, Third Military Medical University (Army Medical University) between May 2017 and February 2020 were retrospectively collected. The selection criteria for patients in this study included (1) male or female patients aged 18–75 years, (2) liver function classified as Child–Pugh class A or B; (3) histologically confirmed hepatocellular carcinoma (HCC) and (4) patients underwent LLR of S8 via a hepatic parenchymal transection-first approach. The following patients were excluded: (1) the presence of severe dysfunction of organs, (2) LLR of S8 via a hepatic parenchymal transection-first approach combined with the resection of other parts of the liver and/or other organs except for cholecystectomy. Our institution instituted a formal multidisciplinary tumor board for the treatment of HCC. All new HCC cases were presented for decision making and discussion. Patients received the whole course of antiviral treatment for Hepatitis B virus (HBV). The prophylactic antibiotic therapy was intravenously administered 30 min before the surgery and maintained until the second postoperative day. Post-operative management included hematischesis, hepatic function protection, analgesia, rehydration and other symptomatic and supportive care.

### Methods

**Fig. 1:**
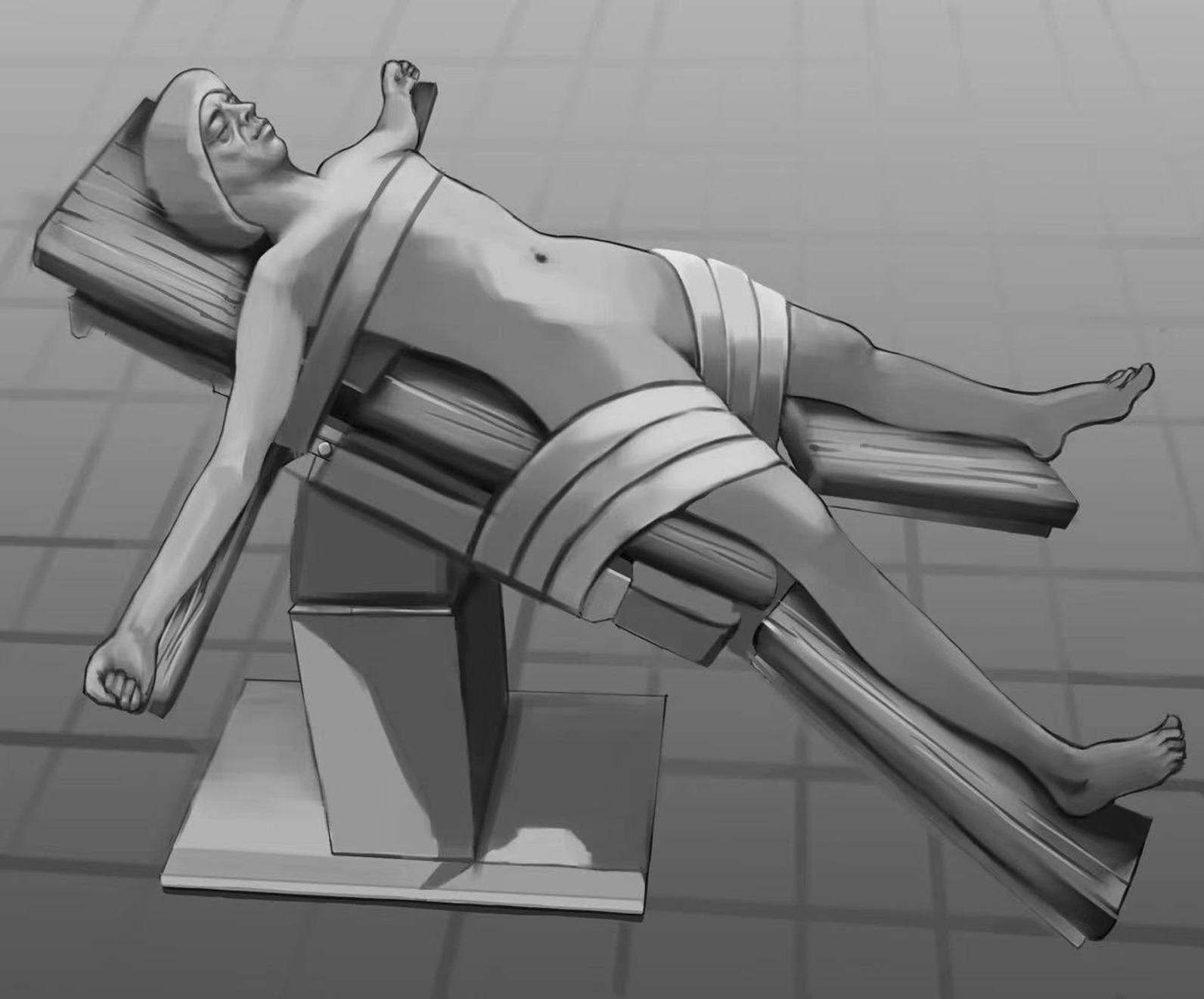
Patient position

**Fig. 2:**
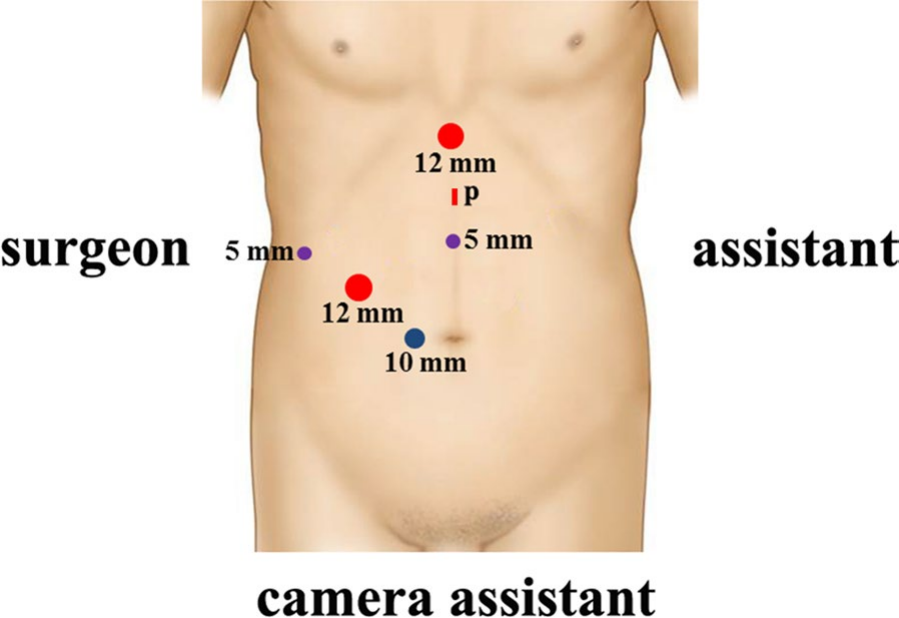
Diagrams of trocar placement for LLR of S8. Two 12-mm trocars, two 5-mm trocars and one 10-mm trocar are used. The incision marked with p was made 3 mm in length for insertion of extracorporeal Pringle’s maneuver

The patient was placed in a reversed Trendelenburg and left semilateral position with head up 30° and leg splitting (Fig. 1). The surgeon stood on the right side of the patient, the camera assistant stood between the spread legs, and the assistant and monitor were on the left side of the patient, facing the surgeon. The trocars were inserted according to the 5-port-method. The initial trocar (10-mm) is placed at a site 2 cm right of the umbilicus and was used as observation port. Two 12-mm ports were inserted at 5 cm below the costal margin on the right midclavicular line (for the right main working) and below the xiphoid (for the left main working), respectively. The 5-mm port was placed at the midpoint of xiphoid process and the umbilicus as the left subsidiary port. Additional 5-mm port was placed at the subcostal area that meets the right anterior axillary line as the right subsidiary port (Fig. 2). A 5-port “J” configuration is established around the target liver segment. This port placement can be placed more cranially according to patient's somatotype, which is intended to facilitate left, caudal and right side transection plane and caudal side transection plane with the transection device. To prepare for extracorporeal Pringle’s maneuver, a 3-mm length incision was made between left two ports through which a self-designed tube would be inserted for holding a cotton tape around the hepatoduodenal ligament. Central venous pressure (CVP) was kept lower than 5 cmH_2_O.

**Fig. 3:**
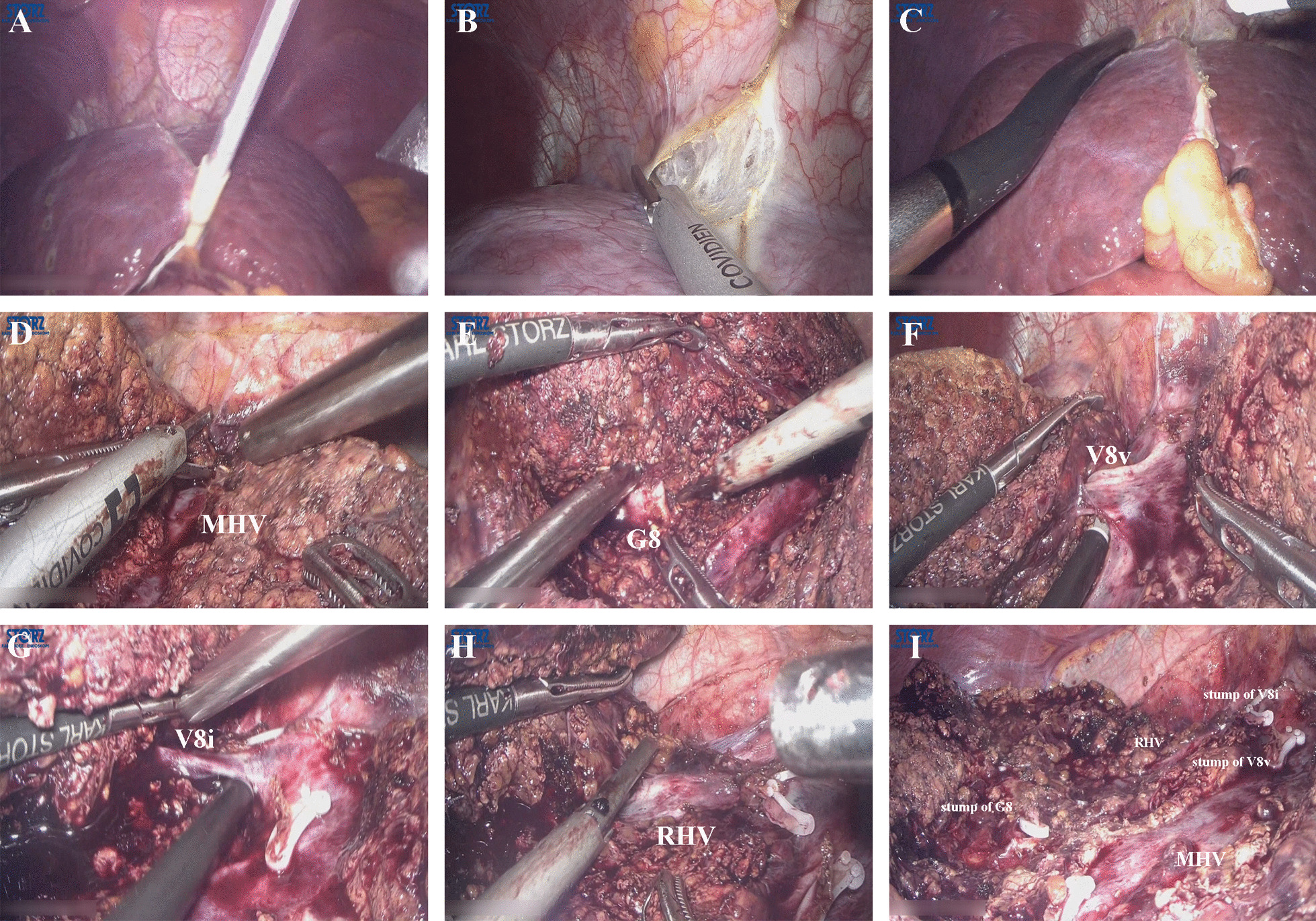
Laparoscopic technique and procedure. **a** Pringle maneuver; **b** Preliminary liver mobilization; **c** IOUS was used to mark the the tumor range and central position and to determine the courses of main trunk of MHV and RHV, and the position of the G8 or G8v and G8d; **d** Prior parenchymal transection along the MHV; **e** Exposing and dividing the G8; **f** Exposing and dividing the V8v; **g** Exposing and dividing the V8i; **h **Liver resection along the RHV toward the caudal direction and then resection completion; **i** Findings after hepatectomy of S8 (Additional file 1)

Intraoperative procedure: (1) Liver mobilization: the round and falciform ligaments were divided to the second hepatic hilum to expose the root of the MHV and right hepatic veins (RHV) and the crypt between MHV and RHV. The right coronary ligament next to the second hepatic hilum was also divided. Resections in s S8 do not usually require a full anti-clockwise rotation of right lobe. After mobilization of the liver, the posterior side of right liver could be well visualized. (2) Preparing for Pringle’s maneuver: Extracorporeal laparoscopic Pringle’s maneuver was prepared. This method can be quickly used in case of major bleeding and declamped easily and fast. (3) Intraoperative laparoscopic ultrasonography (IOUS): IOUS was performed on the liver surface to localize the tumor range and central position and to determine the courses of main trunk of MHV and RHV, and the position of the S8 Glissonean pedicle (G8) or ventral branch of G8 (G8v) and dorsal branch of G8(G8d) and mark them accordingly. (4) Dissection of the left side transection plane and caudal side transection plane liver parenchyma: hepatic resection is begun from the caudal side of the liver at the intersection of the G8 level which was positioned by ultrasound and the Cantlie line via a hepatic parenchymal transection-first approach. The starting point also can be estimated by reference to simulation images constructed using preoperative 3-dimensional reconstruction. The resection is continued from the caudal to cranial side along the markings of MHV (right of the vein), until the critical separation of S8 from liver segment 4a (S4a) was completed. The left side resection plane should be dissected as straight transection lines as these are easier to follow MHV, especially during deeper dissection, allowing the surgeon to evaluate the relationship between the tumor and the intended resection line. Safely exposed hepatic vein branches were clipped and cut. Then, the hepatic resection was continued from the above-depicted starting point toward the right direction of caudal side transection plane and the caudal side liver transection plane parenchyma was divided 1–2 cm for better visualization. The left side transection plane resection and caudal side transection plane resection continued alternately to expose the root of the MHV. S8 was retracted cephalically during this procedure. The resection was continued until the G8 or G8v and G8d were naturally exposed on the resected liver surface. When performing deeper dissection, care must be taken. According to the intraoperative situation, it is possible to repeatedly adjust the transection plane under guidance of IOUS. To reduce air interference in IOUS, saline can be injected on the transection plane. (5) Management of the G8: The caudal side transection plane was further divided toward the dorsal side of the G8 to obtain a large space around the G8. The G8 is clamped. In most cases, the G8 bifurcates into the ventral half and the dorsal half. G8v and G8d were clamped separately. The liver surface ischemic line is marked along the resulting discoloration using electrocautery. Then, the right side transection plane hepatic parenchyma was initially transected along the ischemic line, followed by ligated and divided of the G8 or G8v and G8d. (6) Management of the ventral branch of the draining vein from S8 (V8v) and intermediate vein for S8 (V8i) branches: When hepatic resection is continued further cranially along the MHV, the S8 branches of the MHV, including the V8v and V8i branches and small ducts, are ligated and divided. Titanium clips, hemolock clips or vascular staplers are used to divide progressively larger biliary and vascular structures. (7) Dissection of the right side transection plane liver parenchyma: To expose the root of the RHV, the transection of the liver parenchyma was continued from the root of the MHV toward right side, in the crypt between MHV and RHV and the RHV direction. Subsequently, the RHV is exposed. Then, liver resection is performed toward the caudal direction along the RHV and ischemic line, and the main trunk of the RHV is exposed. At that point, the surgeon was standing at the left side of the patient and performed the parenchyma transection via a cranial approach. Dorsal branch of the draining vein from S8 (V8d), which drains the dorsal portion of S8, is ligated and divided. After the completion of S8, the following structures are exposed at the resected liver surface: MHV, stump of G8, and RHV. (8) Bagging of the resected specimen: A protective bag was inserted intra-abdominally into which the resected specimen was placed. (9) Management of the resection margin: The surgical field was irrigated. The margin was carefully checked for any bleeding or bile leakage. Hemostasis was achieved by using bipolar electrocoagulation or Prolene sutures ligation. Any bile leakage from the suspicious bile ducts was ligated using 5–0 Prolene sutures. Hemostatic products such as fibrillar hemostatic or fibrin glue were used. (10) Specimen removal: After the specimen was resected, the incision for extracorporeal Pringle’s maneuver was extended to the incision for the left subsidiary port for removal of the specimen. (11) Drainage and wound closed: After a silastic drain placed under the right diaphragm, the wound was closed in layers with absorbable sutures (Fig. 3a–i).

### Statistical analysis

Descriptive statistics was used for evaluating variants. Age, operation time, tumor-free margin and follow-up time were expressed as mean ± standard deviation and blood loss and postoperative hospital stay were presented as median and interquartile range. SPSS version 22.0 (IBM SPSS, Inc, Chicago, IL) was used for all analyses.

## Results

**Fig. 4:**
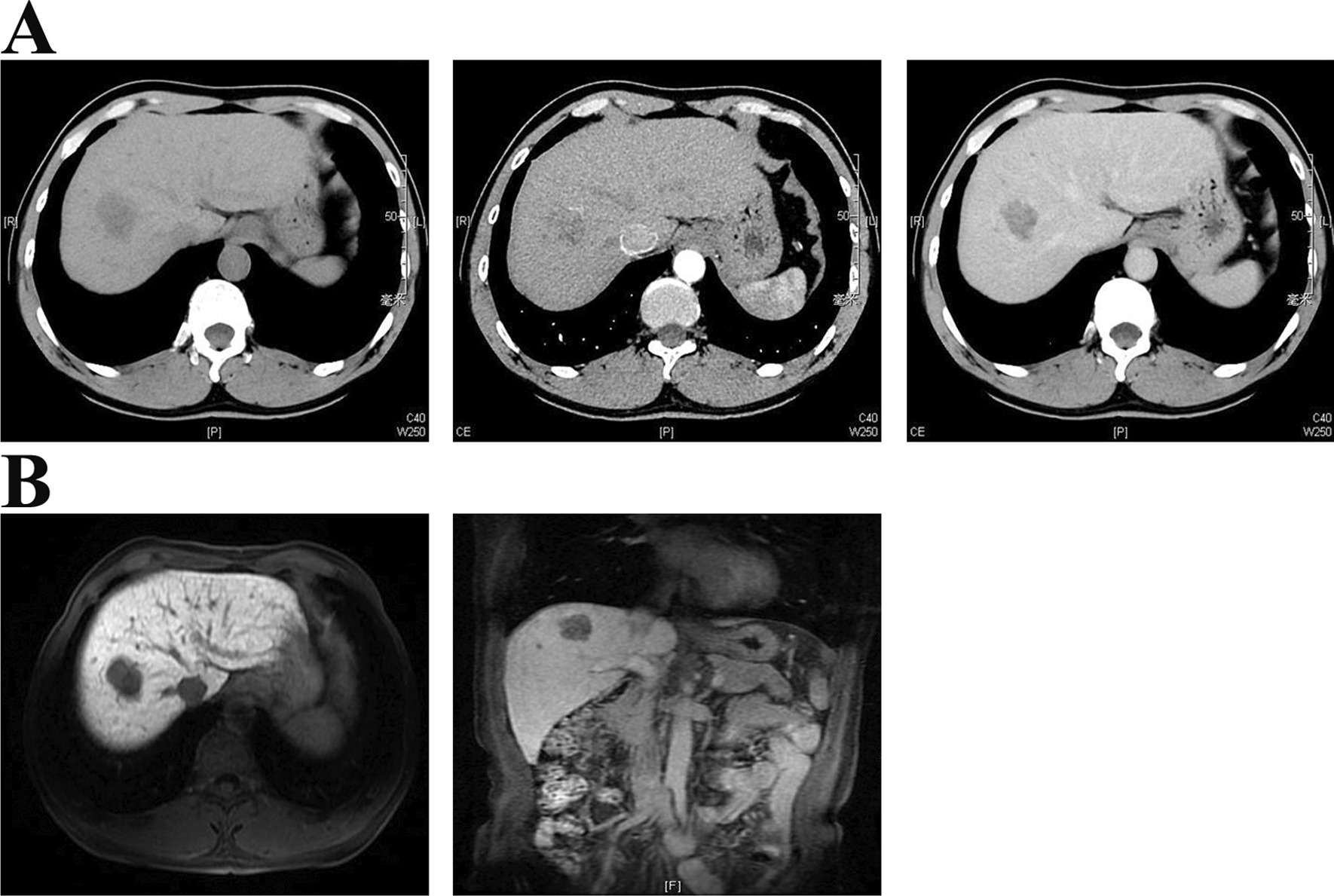
Preoperative CT (**a**) and MRI (**b**) of the liver. A 3.2 cm × 3.0 cm sized mass (arrows) was found on S8

**Fig. 5:**
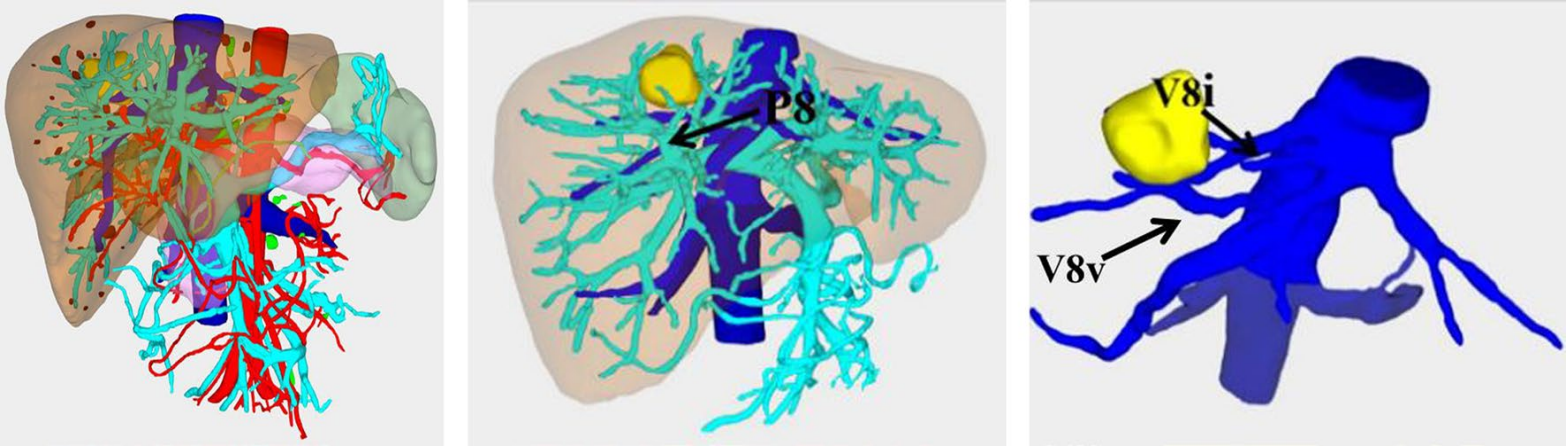
Preoperative 3D-CT reconstruction. The black arrow indicates the G8. 3D-CT reconstruction shows V8v (black arrow) joining the MHV. V8i (black arrow): tributary of MHV running between the ventral part and dorsal part of S8

All 22 patients (mean age 51.1 ± 11.6 years) underwent blood biochemistry and tumor markers analyses, imaging examination (Fig. 4), indocyanine green clearance test, and 3-dimensional reconstruction (Fig. 5) before the operation. After surgery, all patients were diagnosed with HCC. All patients completed the operation successfully without conversion to open surgery. The mean operation time was 186.05 ± 18.4 min, median blood loss was 170.0 ml (143.8–205.0 ml), and blood transfusion was not needed. All patients obtained negative resection margins and the mean tumor-free margin was 10 ± 1.5 mm. The median postoperative hospital stay was 8.0 days (7.0–9.0 days). There was no mortality. Post-operative complications were defined according to Clavien Dindo classification. Two patients had Grade I complications: one liver section effusion and one small amount of pleural effusion. Four patients had Grade II complications: two pneumonia, one intra-abdomen bleeding, treated conservatively with hemostatic drugs and one intra-abdominal collection secondary to bile leak, treated conservatively with the surgical drain already in place from the operation. No patients had Grade III and above complications. All the complications were successfully treated conservatively.

All 22 patients were followed, with a mean follow-up time of 19.6 ± 7.0 months. During the follow-up period, none of them developed hemorrhage, bile leakage, and other complications. There was no reoperation or perioperative mortality during the follow-up. Imaging examination scans showed portal branch of segment 5 (P5) and portal branch of posterior segment (PP) were clearly exposed in almost their entirety and then preserved and the main trunk of the MHV was also preserved. There was no patient had developed tumor recurrence during follow-up period.

## Discussion

In LLR of S8, the main difficulty lies in the choice of surgical approach. The choice of laparoscopic surgical approach for resection of liver S8 is not simply a “road of entry” but a series of strategic decisions on how to accomplish the surgical goals while ensuring the safety and effectiveness of the surgery [8].

To date, the approaches for LLR of S8 include an ultrasound-guided S8 portal branch puncture and localization approach, hepatic hilum Glisson pedicle approach, and left and right hemi-hepatic splitting approach. The ultrasound-guided S8 portal vein puncture and localization approach refers to ultrasound-guided injection of the methylene blue and ICG dye into the S8 portal vein branches to determine the extent of resection during surgery. The puncture is different from the one under ultrasound guidance during open surgery. The existing laparoscopic ultrasound probe and puncture needle are not the best fit for surgeon’s expectations. The intrahepatic portal vein puncture technique under laparoscopic ultrasound requires an experienced hand and specific attention to the puncture angle. Moreover, the portal vein is deeply positioned in S8 and is usually divided into ventral and dorsal branches. Thus, puncturing the corresponding portal veins one by one is difficult, and the borders of the S8 area with dye staining may not display accurately. The above factors lead to a low puncture success rate, and therefore, this approach has been difficult to popularize in a short time [5]. The Glisson pedicle approach refers to removal of S8 after isolation of S8 Glissonean pedicle branch from the right anterior and posterior pedicles. This approach is essentially an extra-Glissonian approach and is safer. However, this approach is difficult and not practical because of the deep location of the S8 Glissonean pedicle, anatomical variation, the small space in the liver hilum area, the difficulty in exposing the hilar plate, the long initial transection plane, the requirements of the dissection technique and the limitations of laparoscopic instruments [9, 10]. This approach is useful and reasonable when S8 Glissonean pedicle ramifies from the right anterior Glissonean pedicle near the hepatic hilum. The left and right hemi-hepatic splitting approach refers to liver resection that is performed from the caudal to cranial side along the Cantlie line, superiorly to the second hepatic hilum and inferiorly to the first hepatic hilum, to complete the mid-split of the liver parenchyma and expose the MHV and then to separate the S8 Glissonian pedicle and remove S8. This approach is associated with a large wound, a risk of biliary leaks and bleeding and may damage right anterior Glissonean pedicle, Glissonean branches of liver segment 5 (S5) and S5 hepatic vein (V5), which increases the difficulty of application [11].

LLR of S8 should follow the principle of “simplification of complicated surgery”. Ome and colleagues found that hepatic parenchyma transection from the root of MHV toward the periphery, called cranial approach is feasible and safe when used in LLR of S8. This approach can avoid complex anatomical separation, accurately determine the transection plane in a simple and convenient manner, and simplify the operation steps [12]. Since the direction of endoscopic view and liver dissection are from the foot to the head side, most surgeons have not got used to perform the cranial approach. Moreover, we believe that the critical problems of cranial approach are thoracic organ injuries and postoperative pneumothorax using intercostal trocars.

We have also carried out related research and explored feasible and safe approach. During laparoscopic right and left hepatectomies, we used a hepatic parenchymal transection-first approach guided by the MHV to control the hepatic Glissonean pedicle and achieved good outcomes [13, 14]. Precise parenchymal transection of the liver is necessary to locate the Glissonean branch of S8 because it is located in the deep parenchyma. Moreover, the main trunk of the MHV is a landmark in resection of liver S8 and the location of the MHV is relatively stable [15, 16]. Therefore, we wondered whether the hepatic parenchymal transection-first approach guided by the MHV can be extended for laparoscopic resection of liver S8. After clinical practice, we proposed laparoscopic resection of liver S8 via the hepatic parenchymal transection-first approach guided by the MHV and made it a standardized and streamlined procedure after continuous exploration and improvement. Different from the cranial approach, our method used caudal approach which makes the operation line with the operation habits of most surgeons. Our method is simple, fast, safe and accurate and has higher clinical application value in laparoscopic resection of liver S8.

**Fig. 6:**
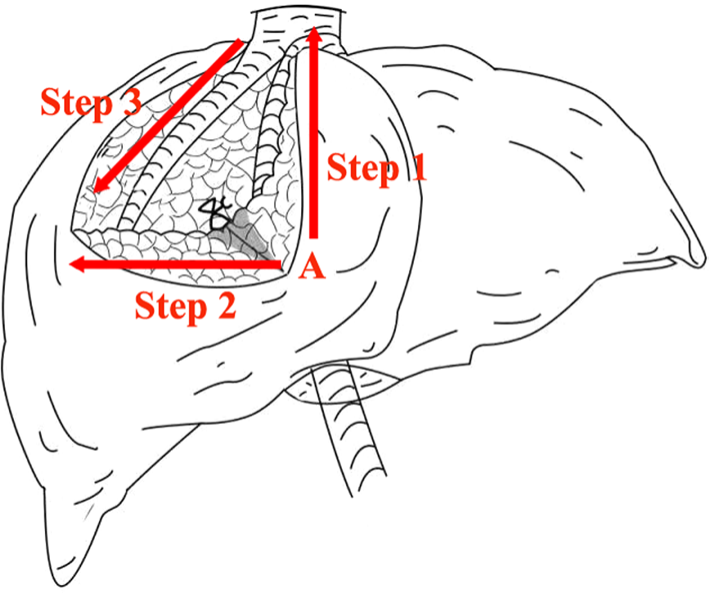
Diagram of hepatic parenchymal transection process. Site A indicates the starting point of the parenchymal dissection. Step 1. Hepatic resection is begun at Site A and continued further cranially along the MHV; and the trunk of the MHV was also exposed; Step 2. Next, the hepatic resection was continued from the Site A toward the right direction of caudal side transection plane; the G8 was exposed and divided; Step 3. Subsequently, the liver parenchyma between S8 and S7 was dissected along the RHV and ischemic line from the root side to the peripheral side; with this, transections finished. In order to full exposure, Step 1 and Step 2 are continued alternately according to the intraoperative situation

The precautions for LLR of S8 via the hepatic parenchymal transection-first approach guided by the MHV are listed as follows: (1) Preoperative high resolution thin-sliced enhanced CT scanning, helical CT arterial portography and 3D reconstruction visualization system can be used to make accurate assessments of the location and courses of Glissonean pedicles, MHV, and RHV in S8 to avoid damaging the vessels that need to be preserved during surgery [17]. (2) This technique is not dependent on liver staining by injection of special dye. Meanwhile, there was no ischemic line to guide parenchyma transected before G8 ligated and divided. Therefore, IOUS is a useful and convenient step to accurately locate important structures and to provide orientation in an area lacking in external landmarks [18]. Without IOUS instrument or lack of technical experience in operating IOUS, a more thorough comprehension of the LLR techniques and imaging data are required. The boundary between S8 and S4a can also be determined by right or left Glissonean pedicle temporary clamping to form ischemic line. During laparoscopic surgery, it is common for surgeons to converge on the specimen; hence, it is important to add an additional 1–2 cm to the resection margins. (3) The starting point of the parenchymal dissection generally starts from the intersection of the S5 and S8 boundaries and the Cantlie line, but during surgery, this point is difficult to determine. To avoid excessive or insufficient parenchymal transection, we generally use the intersection between the caudal side transection plane where the G8 is at and the Cantlie line as the starting point for parenchymal transection. (4) Exposure of the intrahepatic MHV and separation of the G8 are the key steps for success of the laparoscopic resection of liver S8 guided by the MHV. We use IOUS to locate the MHV to determine the left transection plane. The dissection of left side transection plane and the caudal side transection plane are continued alternately (Fig. 6). The liver parenchyma separation does not go deeper than the level of the MHV (the area beyond this is the right caudal lobe), and then, the MHV and the G8 can be exposed in the liver parenchyma. In order to full exposure, partial hepatic parenchyma of S5 and/or S4a could be dissected [19]. (5) During surgery, attention should be paid to not damage the venous that drained S5, which leads to congestion of S5. (6) The management method of G8 should be based on findings of 3D reconstruction visualization system and other imaging assessments to individualize a method. Either branch ligation or main trunk clipping can be used. Control of all structures is essential, not only to reduce the risk of post-operative complications, but to keep the operative field clear to facilitate vision and meticulous dissection. (7) There may be problems, such as inadequate exposure during initial transection of the liver parenchyma. The exposure can be improved by S8 suspension using suture and adjusting patient positioning during the procedure or retracting the liver toward the left and inferior side. The exposure will be sufficient after splitting of the liver parenchyma. (8) When parenchymal transection along the RHV to the caudal side, the surgeon should move from the right side of the patient to the left, and the parenchyma is transected from the cephalic side to the caudal side. Alternatively, two-surgeon technique can be used in which the surgeon and the assistant exchange roles as needed [20].

The clinical value of LLR of S8 via the hepatic parenchymal transection-first approach guided by the MHV is mainly reflected in the following aspects: (1) The resection range can be easily determined with accuracy. (2) Due to special anatomical position and rare anatomic variation in root, the MHV is a good landmark for the hepatic parenchymal transection-first approach, which is direct, convenient, and capable of reducing the length of the initial transection plane. (3) The use of the hepatic parenchymal transection-first approach can avoid the need to perform an elaborate hilar dissection approach, bypassing the surgical obstacle caused by the complicated anatomic variation of hepatic hilar area. By dissection the left and caudal side transection plane of the hepatic parenchyma in relatively nonvascular planes, S8 can be fully lifted cephalically to expose the S8 Glissonean pedicle and MHV. The “dead liver” lacking inflow and outflow could be completely removed. Doing so can avoid damage to blood vessels and bile ducts in the residua liver and reduce the incidence of postoperative complications such as bile leakage, infection, and early tumor recurrence. (4) In our approach, the major hepatic fissure do not need completely divided. The risk of right anterior Glisson pedicle, Glisson branches of S5 and V5 laceration can be avoided [21].

## Conclusion

LLR of S8 is still quite challenging at present, and it is our goal to design a reasonable procedure with accurate efficacy and high safety. We use hepatic parenchymal transection-first approach guided by the MHV for LLR of S8. This technique overcomes the problem of high technical risk, greatly reduces the surgical difficulty and achieves technological breakthroughs, but there are still many problems worth further exploration: (1) The safety of this technique still requires multicenter, large-sample-sized, prospective, randomized controlled studies to verify. (2) Whether selective hemihepatic or total hepatic blood flow occlusion is needed requires continuous improvement based on the actual situation and technological developments. (3) Whether patients with malignant liver tumors will benefit in the long term from this technique is still controversial. Therefore, long-term survival benefits need to be further studied [22].

## Supplementary Information

**Additional file 1**: Video. The operative procedures of LLR of S8 via a hepatic parenchymal transection-first approach guided by the MHV.

## Data Availability

The raw data of the current study are not publicly available due to the protection of participants’ personal information but are available from the corresponding author on reasonable request.

## Abbreviations

CT: Computed tomography
CVP: Central venous pressure
G8: S8 Glissonean pedicle
G8v: Ventral branch of G8
G8d: Dorsal branch of G8
HBV: Hepatitis B virus
HCC: Hepatocellular carcinoma
IOUS: Intraoperative laparoscopic ultrasonography
ICG: Indocyanine green
LLR: Laparoscopic liver resection
MHV: Middle hepatic vein
MRI: Magnetic resonance imagining
P5: Portal branch of segment 5
PP: Portal branch of posterior segment
RHV: Right hepatic veins
S4a: Liver segment
S5: Liver segment 5
S8: Liver segment 8
V5: S5 hepatic vein
V8d: Dorsal branch of the draining vein from S8
V8i: Intermediate vein for S8
V8v: Ventral branch of the draining vein from S8
